# Gradients of structure–function tethering across neocortex

**DOI:** 10.1073/pnas.1903403116

**Published:** 2019-09-30

**Authors:** Bertha Vázquez-Rodríguez, Laura E. Suárez, Ross D. Markello, Golia Shafiei, Casey Paquola, Patric Hagmann, Martijn P. van den Heuvel, Boris C. Bernhardt, R. Nathan Spreng, Bratislav Misic

**Affiliations:** ^a^McConnell Brain Imaging Centre, Montréal Neurological Institute, McGill University, Montréal, QC H3A 2B4, Canada;; ^b^Signal Processing Laboratory (LTS5), Ecole Polytechnique Fédérale de Lausanne, 1015 Lausanne, Switzerland;; ^c^Center for Neurogenomics and Cognitive Research, Vrije Universiteit Amsterdam, 1081 HV, Amsterdam, The Netherlands

**Keywords:** connectome, structure–function, cortical gradient

## Abstract

The white matter architecture of the brain imparts a distinct signature on neuronal coactivation patterns. Interregional projections promote synchrony among distant neuronal populations, giving rise to richly patterned functional networks. A variety of statistical, communication, and biophysical models have been proposed to study the relationship between brain structure and function, but the link is not yet known. In the present report we seek to relate the structural and functional connection profiles of individual brain areas. We apply a simple multilinear model that incorporates information about spatial proximity, routing, and diffusion between brain regions to predict their functional connectivity. We find that structure–function relationships vary markedly across the neocortex. Structure and function correspond closely in unimodal, primary sensory, and motor regions, but diverge in transmodal cortex, particularly the default mode and salience networks. The divergence between structure and function systematically follows functional and cytoarchitectonic hierarchies. Altogether, the present results demonstrate that structural and functional networks do not align uniformly across the brain, but gradually uncouple in higher-order polysensory areas.

## Significance

The white matter architecture of brain networks promotes synchrony among neuronal populations, giving rise to richly patterned functional networks. Relating structure and function is a fundamental question for systems neuroscience, but the nature of the relationship is unknown. Here we examine the possibility that structure–function relationships are not uniform in the brain. We find that structure and function are closely aligned in unimodal cortex (primary sensory and motor regions), but diverge in transmodal cortex (default mode and salience networks). The divergence between structure and function closely follows representational and cytoarchitectonic hierarchies, reflecting a macroscale gradient. Our findings suggest structure and function are not uniformly related, but gradually decouple in parallel to this macroscale gradient.

Intricate connection patterns among neural elements form a complex hierarchical network that promotes signaling and molecular transport (1, 2). Neural elements have a pronounced tendency to form local cliques and tightly coupled communities with common functional properties (3); a small proportion of long-distance projections allows signals to be sampled and integrated from these specialized domains (4–6). Perpetual interactions via the white matter “connectome” manifest as richly patterned neural activity and are thought to support perception, cognition, and action (7).

What is the link between structure and function in brain networks? Relating the organization of physical connections to patterns of functional interactions is a key question in systems neuroscience. A number of methods have been used to address this link, including statistical models (8, 9), communication models (10–12), and biophysical models (13–16). The focus has traditionally been on using whole-brain structural connectivity to predict whole-brain functional connectivity, with the assumption that a common mechanism operates across the entire network. These methods have proved insightful and generally yield moderate fits to empirical functional connectivity patterns, from ∼25% to 50% of the variance explained (17).

Nevertheless, structure and function may not be related in exactly the same way across the whole brain. Recent evidence points to a fundamental organizing principle for macroscale functional interactions (18). Namely, a hierarchy that spans from unimodal primary areas to polysensory transmodal areas traces a continuous sensory-fugal gradient, culminating in the default mode network (19, 20). This representational gradient may reflect microstructural variations, showing significant associations with intracortical myelination (21) and laminar differentiation (22). A prominent account posits that rapid evolutionary expansion of association cortices effectively “untethers” polysensory regions from molecular signaling gradients and canonical sensory-motor activity cascades, resulting in fundamentally different structure–function relationships along the unimodal–transmodal hierarchy (23). Altogether, this work opens the possibility that structure and function may not be related in exactly the same way across the whole brain, but potentially converge or diverge in specific areas.

Here we address the relationship between structure and function by focusing on connection profiles of individual brain regions. We first reconstruct structural and functional networks from diffusion MRI (dMRI) and resting-state functional MRI (fMRI) in a cohort of 40 healthy participants. We then apply a simple multilinear model that uses information about a region’s geometric and structural network embedding to predict its functional network embedding. The method allows us to ask how closely structure and function correspond in individual regions and the extent to which this correspondence reflects affiliation with cognitive systems, cytoarchitecture, and functional hierarchies.

## Results

Structural and functional networks were reconstructed as follows:

### Structural Networks.

Structural and functional connectivities were derived from N=40 healthy control participants (source: Lausanne University Hospital) (24). Structural connectivity was estimated from diffusion spectrum imaging. Adjacency matrices were reconstructed using deterministic streamline tractography. A group-consensus structural connectivity matrix was assembled using a consistency- and length-based procedure (11, 25–27).

### Functional Networks.

Functional connectivity was estimated in the same healthy individuals using resting-state functional MRI (rs-fMRI). Functional connections were defined as zero-lag Pearson correlations among regional time courses. A group-consensus functional connectivity matrix was estimated as the mean connectivity of pairwise connections across individuals.

Initial data exploration was performed at the highest parcellation resolution (1,000 nodes), using group consensus structural and functional networks (see *Materials* and *Methods* for more details). Analyses were subsequently repeated at other resolutions and for individual participants and in an independently collected dataset.

**Fig. 1.**
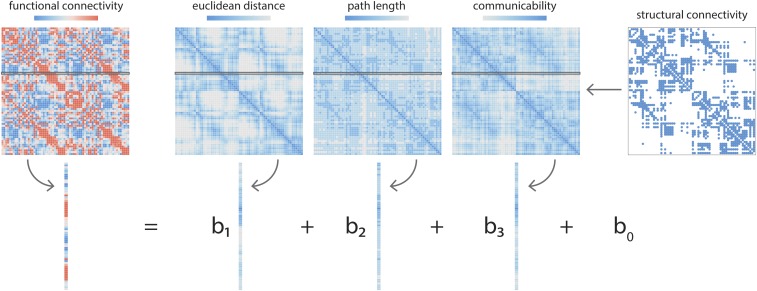
Node-wise structure–function relationships. Local, node-wise structure–function relationships are estimated by fitting a multilinear regression model for each node separately. For a given node i, the response or dependent variable is the functional connectivity between node i and node j ≠ i. The predictor or independent variables are the geometric and structural relationships between i and j, including the Euclidean distance, path length, and communicability. The “observations” are individual i,j relationships. Model parameters (intercept $b_{0}$ and regression coefficients $b_{1}$, $b_{2}$, and $b_{3}$) are then estimated by ordinary least squares. Goodness of fit for each node i is quantified by $R_{i}^{2}$ between observed and predicted functional connectivity.

To estimate the correspondence between local structure and function, we constructed a multilinear regression model that relates node-wise structural and functional connectional profiles (Fig. 1). For a given node i, the dependent variable is the resting-state functional connectivity between node i and all other nodes in the network j ≠ i. The predictor variables are the geometric and structural relationships between i and j, including Euclidean distance, path length, and communicability. The observations or samples are the individual i,j relationships. Model parameters (regression coefficients for each of the 3 predictors) are then estimated via ordinary least squares. Goodness of fit for each node i, representing the correspondence between structural and functional profiles for that node, is quantified by the adjusted $R_{i}^{2}$ between observed and predicted functional connectivity (hereafter referred to simply as $R^{2}$). The use of a multilinear model to relate structure and function is conceptually similar to the method previously reported by Goñi et al. (10) (also refs. 9 and 28), with the important exception that the present model focuses on connection profiles of individual regions rather than whole-brain connectivity.

### Convergent and Divergent Structure–Function Relationships across Neocortex.

**Fig. 2.**
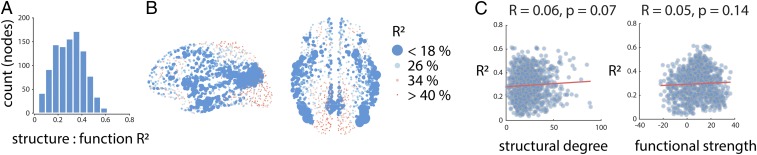
Convergent and divergent structure–function relationships across neocortex. (*A*) Local structure–function correspondence, estimated by node-wise $R^{2}$ from the multilinear model. The histogram shows a wide distribution of $R^{2}$ values across 1,000 nodes at the highest resolution. (*B*) The spatial distribution of structure–function correspondence. Nodes are colored and sized in inverse proportion to $R^{2}$; nodes with weaker structure–function correspondence are larger. High correspondence is observed in primary sensory and motor cortices, while lower correspondence is observed in transmodal cortex. (*C*) Correlation between structural and functional centrality and structure–function correspondence. Shown are scatter plots between node-wise $R^{2}$ and structural and functional centrality, estimated by binary degree and weighted strength, respectively. The low correlations suggest that the correspondence between structure and function does not trivially depend on the structural or functional connectedness of a node. For the same results at other parcellation resolutions, see *SI Appendix*, Fig. S1.

The correspondence between structural and functional connection profiles is highly variable across neocortex. Fig. 2*A* shows the histogram of $R^{2}$ values from each of the node-wise multilinear models. Mean $R^{2}=0.30$ (median $R^{2}=0.30$), roughly concordant with previous reports that used similar models to predict whole-network functional connectivity (10). However, the values vary considerably, from $R^{2}=0.04$ to $R^{2}=0.62$ (interquartile range = 0.18), indicating that for some regions there is a strong correspondence between structural network embedding and function, while for others there is little evidence of any such correspondence.

We next examine the anatomical distribution of structure–function $R^{2}$ values. To highlight regions that show little correspondence, node size and color are inversely proportional to their $R^{2}$ (Fig. 2*B*). The map shows a highly organized and hemispherically symmetric spatial arrangement. Brain regions with least structure–function correspondence include medial parietal structures (precuneus, posterior cingulate), lateral parietal and temporal cortices, insular cortex, and anterior cingulate cortex. Conversely, primary sensory regions, including occipital and paracentral cortices, show relatively high structure–function correspondence.

It is possible that low $R^{2}$ values are observed in some areas because they have either too many or too few direct connections. To examine this possibility, we correlated regional $R^{2}$ values with the structural degree and functional strength of each node (Fig. 2*C*). In both cases the correlations were low (structural, R=0.06, P=0.07; functional, R=0.05, P=0.14), suggesting that regional variations in structure–function correspondence were not trivially driven by structural or functional centrality. We subsequently repeated these analyses for all 5 resolutions of the Lausanne atlas. The results are shown in *SI Appendix*, Fig. S1 and are consistent across resolutions. We also replicated these findings in an independently collected dataset at resolutions 2, 3, and 4 (Human Connectome Project; *SI Appendix*, Fig. S2). The spatial patterns of $R^{2}$ values are visually similar (*SI Appendix*, Fig. S2*A*) and significantly correlated (*r* = 0.77, P=0.0001; 0.72, $P<10^{-4}$; and 0.67, $P<10^{-4}$; *SI Appendix*, Fig. S2*B*).

Finally, structure–function $R^{2}$ values were benchmarked against 3 populations of surrogate networks (*SI Appendix*, Fig. S3). Surrogate networks included rewired networks that randomize both topology and geometry (29), repositioned networks that randomize geometry but not topology (30), and cost-neutral rewired networks that randomize topology but not geometry (6, 31, 32). In all cases, the structure–function $R^{2}$ in the empirical networks significantly exceeded the null $R^{2}$ values observed in the surrogate networks.

### Structure–Function Relationships Follow Functional and Cytoarchitectonic Hierarchies.

The spatial distribution of $R^{2}$ values suggests that structure–function correspondence may be circumscribed by functional systems or cytoarchitectonic attributes. To address this question, we applied 2 partitions: 1) resting-state networks described by Yeo et al. (33) and 2) cytoarchitectonic classes described by von Economo and Koskinas (34–36). The former groups brain regions according to how similar their time courses are and the latter groups regions according to how similar they are in terms of cell morphology.

We first calculated the mean $R^{2}$ for each network or class. To assess the extent to which these means are determined by the partition and not trivial differences in size, coverage, or symmetry, we used a spherical projection null model that permutes class positions while preserving spatial autocorrelation (37). Network or class labels were randomly rotated and mean $R^{2}$ values were recomputed (10,000 repetitions). The network- or class-specific mean $R^{2}$ was then expressed as a z score relative to this null distribution, which we refer to as the “spin test” throughout the rest of this report.

**Fig. 3.**
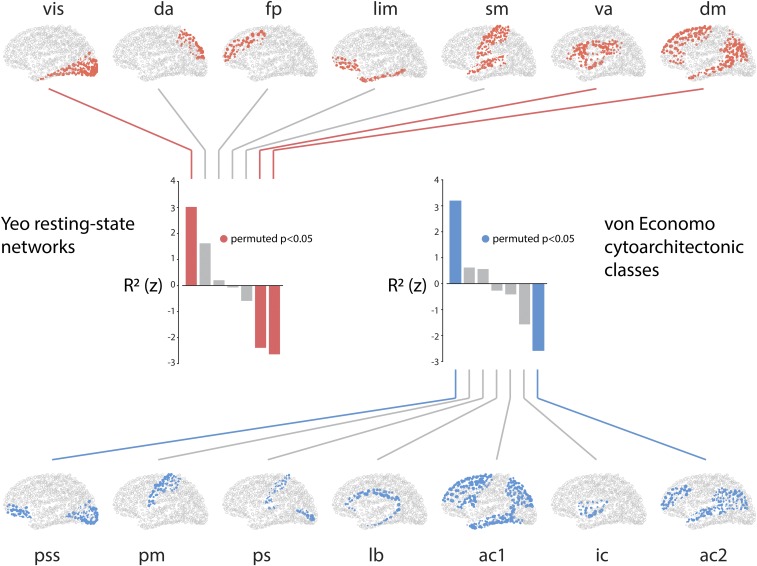
Structure–function tethering across cognitive systems and cytoarchitectonic classes. Node-wise $R^{2}$ values are averaged according to their membership in resting-state networks or cytoarchitectonic classes. To determine whether the mean value for each network or class is statistically significant, a null distribution is constructed by spherical projection and rotation (10,000 repetitions). The network- or class-specific mean $R^{2}$ is then expressed as a z score relative to this null distribution. Statistically significant networks/classes are shown in color; nonsignificant networks/classes are shown in gray. Yeo networks: da, dorsal attention; dm, default mode; fp, frontoparietal; lim, limbic; sm, somatomotor; va, ventral attention; vis, visual. von Economo classes: ac1, association cortex; ac2, association cortex; ic, insular cortex; lb, limbic regions; pm, primary motor cortex; ps, primary sensory cortex; pss, primary/secondary sensory.

There is a gradual divergence between structure and function moving from unimodal to transmodal cortex. Fig. 3 shows the z-scored $R^{2}$ for each resting-state network (red) and cytoarchitectonic class (blue). Positive values indicate that the structure–function relationship is stronger than expected by chance, while negative values indicate that the structure–function relationship is weaker than expected by chance. Consistent with the intuition developed in the previous section, statistically significant divergence between structure and function is observed in polysensory or transmodal cortex, namely the default mode (P=0.0006) and ventral attention (P=0.012) networks and association cortex class (ac2; P=0.0001). The reverse is true for primary unimodal cortex, where there is a significant convergence between structure and function (visual network, P=0.001; primary/secondary sensory class, $P<10^{-4}$).

### Structure and Function Systematically Diverge along a Macroscale Functional Gradient.

Recent studies suggest a universal organizational principle whereby brain regions are situated along a continuous gradient or hierarchy, ranging from primary sensory and motor regions to transmodal regions (18, 38). It is therefore possible that the patterns of structure–function convergence and divergence recapitulate this hierarchy.

**Fig. 4.**
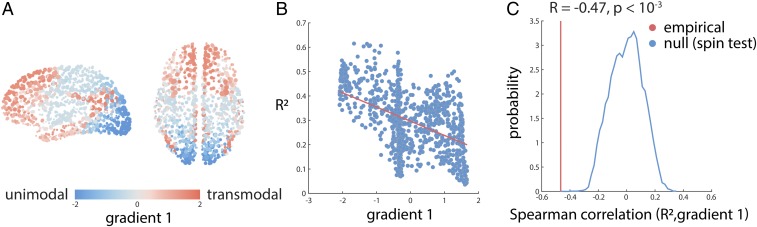
Structure–function divergence across large-scale functional network gradients. Large-scale functional network gradients were identified by applying diffusion map embedding to the normalized graph Laplacian of the correlation matrix. (*A*) The first gradient runs from primary, unimodal cortex to transmodal cortex and resembles the vertex-wise map originally reported by Margulies et al. (18). (*B*) Node-wise structure–function $R^{2}$ values are anticorrelated with positions along this gradient, suggesting that structure and function closely correspond in unimodal cortex but diverge in transmodal cortex. (*C*) Red: empirical Spearman correlation between the first gradient and structure–function $R^{2}$ values. Blue: null distribution of Spearman correlation coefficients derived using spherical projections.

We first derived a macroscale functional gradient for the present dataset. The correlation-based functional network was converted to a transition probability matrix and subjected to singular-value decomposition, a method known as diffusion map embedding (39) (see *Materials* and *Methods* for more details). The first eigenvector of the matrix, which we refer to as a “gradient,” spans primary unimodal cortex on one end and transmodal cortex on the other (Fig. 4*A*). Critically, the map bears a strong resemblance to the vertex-wise map originally reported by Margulies et al. (18).

We then assess the relationship between structure–function $R^{2}$ for a given region and its position along the macroscale functional gradient. Fig. 4*B* shows that the 2 are anticorrelated (Spearman R=−0.48). Comparison to an autocorrelation-preserving spherical projection null model demonstrates that the anticorrelation is statistically significant (37) (Fig. 4*C*; $P<10^{-4}$). In other words, structure and function closely correspond in unimodal cortex, but diverge as one moves up the hierarchy. At the apex of the hierarchy (transmodal cortex), there is much less correspondence between structural and functional connection profiles.

### Alternative Predictors and Individual Participants.

As a final step, we ask 2 important questions. First, how sensitive are the overall results to choice of predictors? Thus far, we focused on 2 canonical network metrics, one related to shortest-path routing and the other related to diffusion. Although theoretically driven, the choice of these 2 measures is arbitrary and there exist several other network-theoretic statistics that also capture the potential for 2 nodes to exchange signals with each other (40), including alternative forms of diffusion (10, 41, 42), contagion (11), parallel exchange via path ensembles (43), and navigation (30).

We therefore repeated the analysis shown above using a multilinear model with a greater number of predictors [Euclidean distance, path length, and communicability as before and adding search information, path transitivity (10, 44–46)]. We note 2 key results. First, the overall model fit does not change appreciably, with the mean $R^{2}=0.33$ (median $R^{2}=0.33$ and SD = 0.12). This is unsurprising, given the well-known multicollinearity among graph measures (47). More importantly, the spatial distribution of $R^{2}$ values is highly correlated with those produced by a multilinear model with fewer predictors (R=0.98, $P<10^{-5}$), suggesting little practical benefit for including additional predictors. The relative contribution of each variable, estimated using single-predictor $R^{2}$, stepwise regression, and standardized β values, is shown in *SI Appendix*, Fig. S4.

The second question is, To what extent can comparable effects be observed in individual participants? In an effort to amplify the signal-to-noise ratio we initially performed all analyses on group-representative structural and functional networks, and it is unclear whether the systematic divergence between structure and function is robust across individuals. We therefore fitted a multilinear model to each individual participant and estimated regional $R^{2}$ values as before. We then correlated the individual-level $R^{2}$ pattern with the group-level $R^{2}$ pattern. The individual-to-group correlation R is moderate (mean R=0.33, median R=0.32, 95% CI [0.07 0.50]), but statistically significant (P<0.05 in 36/40 participants), suggesting considerable consistency across individuals.

### Disentangling Topology and Geometry.

Given that the Euclidean distance is the single most informative predictor by multiple criteria (*SI Appendix*, Figs. S3 and S4), we sought to disentangle the contribution of topology and geometry to the prediction of functional connectivity. *SI Appendix*, Fig. S5*A* shows the distribution of $R^{2}$ values for models using only topological or geometric predictors (blue) vs. the model using all predictors (red). In both cases, the distribution is shifted to the left, suggesting that both topology and geometry contribute to prediction of functional connectivity. *SI Appendix*, Fig. S5*B* shows node-wise $R^{2}$ values of pure topological and geometric models against the combined model, with the identity line shown in red. In both cases, the $R^{2}$ values are correlated but the point clouds lie below the identity line, suggesting that the combined model outperforms the topological and geometric models for most nodes. Finally, *SI Appendix*, Fig. S5*C* shows the node-wise $R^{2}$ values for the geometric and topological models, demonstrating that the geometric model yields greater prediction for 696/1,000 nodes (69.6%), while the topological model yields greater prediction for 304/100 nodes (30.4%).

Given the prominence of spatial proximity as a predictor, it is possible that connection profiles of unimodal areas are more spatially constrained, while transmodal areas are more spatially distributed (48). Although we ruled out the possibility that regional differences in total structural degree or functional strength could be driving the observed patterns (Fig. 2*C*), we pursued the idea that there could be regional variation in connection length and density. For each node, we constructed a histogram of connection lengths to other nodes in the network (10 equal-width bins, spanning 0 to 170 mm). We then plotted these histograms for both structural connectivity (the number of structural connections) and functional connectivity (mean correlation) (*SI Appendix*, Fig. S6). Nodes are ordered by their position along the unimodal–transmodal axis or gradient. The key observation is that there is no systematic interaction between gradient position, connection length, and connection strength.

## Discussion

The present report demonstrates variation in the extent to which structure and function correspond in human cortical networks. The relationship between structural and functional connection profiles appears to follow an overarching cognitive-representational and cytoarchitectural hierarchy, becoming increasingly untethered as one moves toward the transmodal cortex at the apex.

### Localized Structure–Function Relationships.

Our results contribute to a growing effort to understand structure and function from a more localized perspective. There is a rich literature on predicting function from structure at the whole-network level, including direct edge-to-edge comparisons (49, 50), multivariate statistical models (8, 9), network-theoretic models (10–12, 30, 51), and biophysical models (14–16). We find that the relationship is not uniform throughout the whole network, but may instead vary across brain regions. This is consistent with the notion that individual areas possess distinct connectional (52, 53) and spectral activation “fingerprints” (54). High-density precision mapping studies suggest that functional organization and regional boundaries may also be highly individualized (55, 56).

For the present analysis we chose 2 predictors that cover the extremes of a putative communication spectrum (40), one reflecting routing of information and the other reflecting diffusion. The extent to which signaling is centralized or decentralized is an exciting open question (30, 57). For instance, individual areas may broadcast information differently from one another, while large-scale systems may utilize different forwarding protocols or frequency channels (58). Our results open the possibility that communication mechanisms may be multiplexed, with multiple protocols operating in parallel (10).

It is noteworthy that simple Euclidean distance was a powerful predictor. The probability of structural connectivity (31, 59) and the magnitude of functional connectivity between areas both decrease with spatial separation (60, 61). Indeed, many topological attributes of brain networks can be accounted for by simple generative mechanisms that minimize interareal wiring cost (62, 63) (also refs. 27 and 64). Our results are consistent with this notion, showing that the spatial embedding of brain regions is the most informative predictor of their functional interactions.

The prominence of geometry raises the possibility that systematic deviations between structure and function could be driven by differences in distances spanned by individual regional connection fingerprints (6, 48). For instance, transmodal association networks are typically more spatially distributed than unimodal networks, resulting in more spatially distributed connection patterns. Thus, spatial proximity may better explain functional connection patterns in unimodal networks compared to transmodal networks. *SI Appendix*, Fig. S4 supports this possibility, showing that spatial proximity greatly contributes to the structure–function correspondence in the visual system. At the same time, we find little evidence of systematic regional differences in structural or functional connection length and density profiles (*SI Appendix*, Fig. S6). Altogether, the present results demonstrate that spatial proximity makes an important contribution to structure–function relationships, making the deviation between structure and function in transmodal areas all the more interesting.

### Functional and Cytoarchitectonic Hierarchies.

More generally, our findings contribute to an emerging literature that emphasizes macroscopic spatial gradients as a primary organizing principle (19, 20, 38, 65). Smooth variation across cortex has been observed in gene expression (66), cytoarchitecture (67), myeloarchitecture (21), cortical thickness (68), structural connectivity (69), and functional connectivity (18). The increasing complexity of cortical microcircuitry along this hierarchical gradient, ranging from primary sensory to transmodal cortex, is thought to support increasingly integrative internal representations and functions.

Our findings suggest that a consequence of hierarchical microscale organization is a gradual decoupling of macroscale structure and function. In primary sensory areas we find a close correspondence between structural and functional connection profiles, but at the apex of the hierarchy—corresponding to the default mode and salience networks—the 2 diverge considerably. The polyfunctional hubs that occupy this end of the gradient are thus more likely to participate in multiple networks and explore a wider dynamic repertoire (70, 71). How the correspondence between node-level structure and function relates to individual differences in behavior is an exciting question for future work (72). A related question is whether structure and function remain untethered during task performance.

Why do structural and functional networks come untethered? One theory posits that rapid evolutionary expansion of the cortical mantle may have shifted underlying microcircuit organization away from serial bottom–up activity cascades in primary sensory-motor regions (23). Filling the gaps between these primary sensory-motor anchors are association areas marked by parallel and reentrant pathways, lacking obvious hierarchical organization (73). This reconfiguration effectively releases the association cortex from strong constraints imposed by early activity cascades (23), promoting polysensory integration (19), control, and higher cognitive functions that transcend simple sensory-motor exchange, such as internal mentation.

The reconfiguration of local microcircuitry may thus contribute to greater signal variance in transmodal cortex. In particular, shifts in structure–function relationships mirror patterns of laminar differentiation (20, 22). In primary areas with strong differentiation there is a strong correspondence between structure and function, while in transmodal cortex—with weaker laminar differentiation—the structure–function relationship is also weaker. In a recent modeling study, Wang et al. (74) allowed microscale-related parameters of a biophysical model to differ between brain regions. The best-fitting model was characterized by strong recurrent connections and excitatory subcortical input in sensorimotor regions; conversely, default network regions had weak recurrent connections and excitatory subcortical inputs (74). Complementary results were reported by Demirtaş et al. (75), who found that biophysical models could be fitted to functional connectivity much better if they were informed by hierarchical heterogeneity, estimated from T1w/T2w ratios. Thus, a richer local cytoarchitecture in transmodal cortex—supporting increasingly autonomous and spontaneous dynamics—may potentially render macroscale structural metrics less effective overall in predicting functional interactions (76).

Indeed, one explanation for the observed divergence of structure and function could be that the 2 are related but that the methods typically used to define structural and functional connectivity may obscure such relationships in transmodal areas. For example, the conventional static and dyadic representation of functional connectivity employed here may not adequately capture the dynamic character of functional interactions (77) or the possibility of higher-order interactions (78). We have previously suggested that the network embedding of polysensory association areas places them in an optimal position to simultaneously receive signals originating from multiple sources across the network (11, 79). Thus, extensive mixing of diverse signals at the top of the hierarchy may engender less predictable functional relationships and wider discrepancy between structure and function.

### Methodological Considerations.

The present results are subject to several important methodological limitations and considerations. First, structural connectivity is estimated using streamline tractography on diffusion-weighted imaging, a method known to be susceptible to systematic false positives and false negatives (80–82). For instance, many transmodal regions identified as having low structure–function correspondence occupy lateral positions and are known to participate in multiple transcallosal fibers. In addition, diffusion-weighted imaging may create systematically uncertain estimates of structural connectivity in regions with highly developed supragranular layers (such as AC2) or in agranular cortex (such as I). It is therefore possible that regional variation in structure–function correspondence is partly explained by regional variation in tractography performance.

A similar set of concerns exists for reconstruction of functional networks. For example, previous reports have found evidence that functional connectivity may also be more variable in heteromodal or transmodal cortex (83). Moreover, we operationalized functional connectivity in terms of conventional linear zero-lag Pearson correlations, eschewing the possibility that transmodal regions participate in more complex, higher-order interactions. It is also possible that the scrubbing procedure used to control motion artifact could potentially suppress true functional interactions and influence regional difference in structure–function coupling. Altogether, it is possible that there exist prominent structure–function relationships in transmodal cortex, but that the present methodology systematically underrepresents or mischaracterizes structural and/or functional connectivity in these regions, manifesting as a lower correspondence between structural and functional connectivity.

Another concern is that our results are based on parcellated data, a methodological approach that assumes that brain regions can be mapped to identical spatial locations in every participant. Recent evidence from precision-mapping studies, using repeated measurements in single individuals, suggests that functional boundaries can systematically vary across individuals and that this is particularly true in higher-order, transmodal cortex (55, 56).

Finally, it is important to acknowledge that the present multilinear model or models violate a basic assumption of regression models, namely that the observations (regional connection profiles) are not independent. Each observation represents a dyadic (i,j) relationship that is drawn from a graph that represents the brain, a system we know to be spatially contiguous and assume to be connected. The expected effect is that parameter estimates and goodness-of-fit metrics will therefore be biased. For this reason, we only use structure–function $R^{2}$ as a relative metric to compare the correspondence of structure and function across a set of nodes, each of which is estimated under the same conditions.

## Materials and Methods

### Data Acquisition.

We performed all analyses in 2 datasets. The main (discovery) dataset was collected at the Department of Radiology, University Hospital Center and University of Lausanne (LAU) (*n* = 40). We also included a replication cohort from the Human Connectome Project (HCP) (*n* = 215) (84). Structural connectivity was reconstructed from diffusion-weighted imaging: diffusion spectrum imaging (DSI) for LAU and high-angular resolution diffusion imaging (HARDI) for HCP. Although dataset LAU had fewer participants, we selected it as the discovery dataset because of the quality of the DSI sequence. Below we describe the acquisition, processing, and connectome reconstruction procedure for each dataset in more detail.

#### LAU.

A total of *n* = 40 healthy young adults (16 females, 25.3±4.9 y old) were scanned at the Department of Radiology, University Hospital Center and University of Lausanne. Informed consent was obtained from all participants and the protocol was approved by the Ethics Committee of Clinical Research of the Faculty of Biology and Medicine, University of Lausanne. The scans were performed in a 3-T MRI scanner (Trio; Siemens Medical), using a 32-channel head coil. The protocol included 1) a magnetization-prepared rapid acquisition gradient echo (MPRAGE) sequence sensitive to white/gray matter contrast (1 mm in-plane resolution, 1.2 mm slice thickness), 2) a DSI sequence (128 diffusion-weighted volumes and a single b0 volume, maximum b-value 8,000 ${s/mm}^{2}$, 2.2×2.2×3.0 mm voxel size), and 3) a gradient echo echo-planar imaging (EPI) sequence sensitive to blood-oxygen-level-dependent (BOLD) contrast (3.3 mm in-plane resolution and slice thickness with a 0.3-mm gap, TR 1,920 ms, resulting in 280 images per participant). Participants were not subject to any overt task demands during the fMRI scan.

#### HCP.

A total of *n* = 215 healthy young adults (112 females, 29.7±3.4 y old) were scanned as part of the HCP Q3 release (84, 85). Participant recruitment procedures and informed consent forms, including consent to share deidentified data, were previously approved by the Washington University Institutional Review Board as part of the HCP. MRI data were acquired on the HCP’s custom 3-T Siemens Skyra with a 32-channel head coil. The protocol included 1) a 3D-MPRAGE sequence, 2) a HARDI sequence, and 3) a multiband accelerated 2D-BOLD EPI sequence sensitive to BOLD contrast. For more details regarding the acquisition protocol see refs. 84 and 85.

### Structural Network Reconstruction.

Gray matter was parcellated into 68 cortical nodes according to the Desikan–Killiany atlas (86). These regions of interest were then further divided into 4 additional, increasingly finer-grained resolutions, comprising 114, 219, 448, and 1,000 approximately equally sized nodes (87). Structural connectivity was estimated for individual participants using deterministic streamline tractography. The procedure was implemented in the Connectome Mapping Toolkit (88), initiating 32 streamline propagations per diffusion direction for each white matter voxel.

To mitigate concerns about inconsistencies in reconstruction of individual participant connectomes (89, 90), as well as the sensitive dependence of network measures on false positives and false negatives (81), we adopted a group-consensus approach (25, 63, 80). In constructing a consensus adjacency matrix, we sought to preserve 1) the density and 2) the edge-length distribution of the individual participant matrices (11, 25, 27).

We first collated the extant edges in the individual participant matrices and binned them according to length. The number of bins was determined heuristically, as the square root of the mean binary density across participants. The most frequently occurring edges were then selected for each bin. If the mean number of edges across participants in a particular bin is equal to k, we selected the k edges of that length that occur most frequently across participants. To ensure that interhemispheric edges are not underrepresented, we carried out this procedure separately for inter- and intrahemispheric edges. The binary densities for the final whole-brain matrices were 28.1%, 20.3%, 12.0%, 5.9%, and 2.4% for resolutions 1 to 5, respectively.

### Functional Network Reconstruction.

Functional MRI data were preprocessed using procedures designed to facilitate subsequent network exploration (91). fMRI volumes were corrected for physiological variables, including regression of white matter, cerebrospinal fluid, and motion (3 translations and 3 rotations, estimated by rigid body coregistration). BOLD time series were then subjected to a low-pass filter (temporal Gaussian filter with full width at half maximum equal to 1.92 s). The first 4 time points were excluded from subsequent analysis to allow the time series to stabilize. Motion “scrubbing” was performed as described by Power et al. (91). The data were parcellated according to the same atlas used for structural networks (87). Individual functional connectivity matrices were defined as zero-lag Pearson correlation among the fMRI BOLD time series. A group-consensus functional connectivity matrix was estimated as the mean connectivity of pairwise connections across individuals.

### Multilinear Model.

A multiple-regression model was used to predict the functional connection profile of every node, using a set of geometric and structural connection profile predictors of the same node (Fig. 1). The predictors were 1) the Euclidean distance between node centroids, 2) path length between nodes, and 3) communicability between nodes. Path length and communicability were both estimated from the binarized structural connectome. Path length refers to the shortest contiguous sequence of edges between 2 nodes. Communicability ($C_{ij}$) between 2 nodes i and j is defined as the weighted sum of all paths and walks between those nodes (92). For a binary adjacency matrix A, communicability is defined as

$$C_{ij}=\overset{\infty }{\underset{n=0}{\sum }}\frac{{\left[A^{n}\right]}_{ij}}{n!}={\left[e^{A}\right]}_{ij}$$ [1]

with walks of length n normalized by n!, ensuring that shorter, more direct walks contribute more than longer walks. Path length was implemented using the Brain Connectivity Toolbox (https://sites.google.com/site/bctnet/) (93).

The regression model was then constructed for each node i,

$$FC_{i}=b_{0}+b_{1}EU_{i}+b_{2}PL_{i}+b_{3}CO_{i},$$ [2]

where the response variable $FC_{i}$ is the set of functional connections between i and all other nodes, and the predictor variables are the Euclidean distance ($EU_{i}$), structural path length ($PL_{i}$), and structural communicability ($CO_{i}$) between i and all other nodes in the network. The regression coefficients $b_{1}$, $b_{2}$, and $b_{3}$, as well as the intercept $b_{0}$, were then solved by ordinary least squares (function *fitlm.m* in MATLAB 2016a).

### Diffusion Map Embedding.

Diffusion map embedding is a nonlinear dimensionality reduction algorithm (39). The algorithm seeks to project a set of embeddings into a lower-dimensional Euclidean space. Briefly, the similarity matrix among a set of points (in our case, the correlation matrix representing functional connectivity) is treated as a graph, and the goal of the procedure is to identify points that are proximal to one another on the graph. In other words, 2 points are close together if there are many relatively short paths connecting them. A diffusion operator, representing an ergodic Markov chain on the network, is formed by taking the normalized graph Laplacian of the matrix. The new coordinate space is described by the eigenvectors of the diffusion operator. In keeping with previous reports that applied the method to functional networks, we set the diffusion rate α=0.5 (18, 94), which approximates the Fokker–Planck diffusion. The procedure was implemented using the Dimensionality Reduction Toolbox (https://lvdmaaten.github.io/drtoolbox/) (95).

### Null Models.

To assess the relationship between regional structure–function $R^{2}$ values and network membership or gradient position, we applied a recently developed null model that preserves the spatial embedding and autocorrelation of our parcellations (37). We first created a surface-based representation of our parcellations by applying the Lausanne atlas to the FreeSurfer *fsaverage* surface using files obtained from the Connectome Mapper toolkit (https://github.com/LTS5/cmp) (88). We used the spherical projection of the *fsaverage* surface to define spatial coordinates for each parcel by selecting the vertex closest to the center of mass of each parcel. Vertices were projected to a sphere, randomly rotated, and reassigned to the closest parcel (10,000 repetitions). The procedure was performed for one hemisphere and mirrored in the other, as proscribed by Alexander-Bloch et al. (37). The procedure was performed at the parcel resolution rather than the vertex resolution to avoid up-sampling the structure–function $R^{2}$ statistic, which is estimated at the parcel level.

Regional structure–function $R^{2}$ values were additionally benchmarked against 3 populations of null networks (10,000 repetitions each): rewired networks, cost-neutral rewired networks, and repositioned networks. Rewired networks were generated by randomly swapping pairs of edges (10 swaps per edge). The procedure preserves network size, density, and degree sequence, but systematically destroys network topology (29). Cost-neutral rewired networks additionally preserve the edge-length distribution (i.e., geometry) of the network, (6, 31, 32). Finally, spatially repositioned networks were generated by randomly permuting the x, y, z location of network nodes, disrupting the geometry of the network but preserving its topology (30). The first 2 models embody the null hypothesis that network statistics (e.g., regional SC-FC $R^{2}$) do not depend on network topology. The last model embodies the null hypothesis that network statistics do not depend on network geometry/spatial embedding.

### Data Availability.

The Lausanne dataset is available at https://zenodo.org/record/2872624#.XOJqE99fhmM (24). The Human Connectome project dataset is available at https://www.humanconnectome.org/study/hcp-young-adult. Code used to conduct the reported analyses is available at https://github.com/netneurolab or https://doi.org/10.5281/zenodo.2580387.

## Supplementary Material

Supplementary File
