# Supplementary material for: Gradients of structure–function tethering across neocortex
Source: Proc Natl Acad Sci U S A. 2019 Sep 30;116(42):21219–27. doi: 10.1073/pnas.1903403116 (PMC6800358; doi:10.1073/pnas.1903403116)
Supplement: Supplementary File [file pnas.1903403116.sapp.pdf]

PNAS
www.pnas.org

Supplementary Information for

Gradients of structure-function tethering across neocortex

Bertha Vázquez-Rodríguez, Laura E. Suárez, Golia Shafiei, Ross D. Markello, Casey Paquola, Patric Hagmann, Martijn P. van den Heuvel, Boris C. Bernhardt, R. Nathan Spreng, Bratislav Misic

Bratislav Misic.
E-mail: bratislav.misic@mcgill.ca

This PDF file includes:

Figs. S1 to S6

www.pnas.org/cgi/doi/10.1073/pnas.1903403116

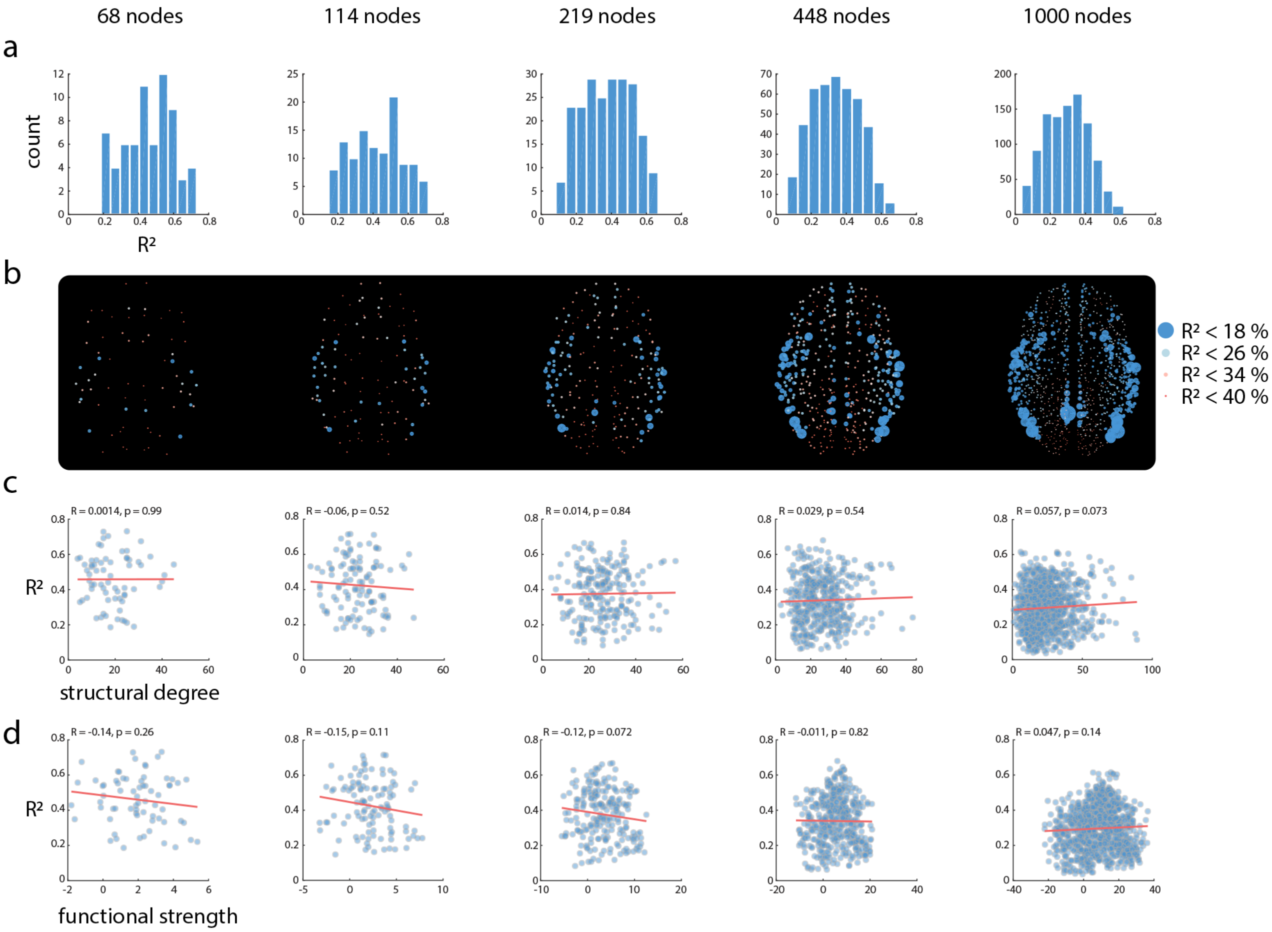

Fig. S1. Stability of main results across five parcellations | The results shown in Fig. 2 are repeated for five anatomical parcellations, featuring 68, 114, 219, 448 and 1000 cortical nodes. (a) Histograms of node-wise R^2 values from the structure-function multilinear model. (b) Spatial distributions of R^2 values. (c) Correlations between node structural degree and R^2 values. (d) Correlations between node functional strength and R^2 values.

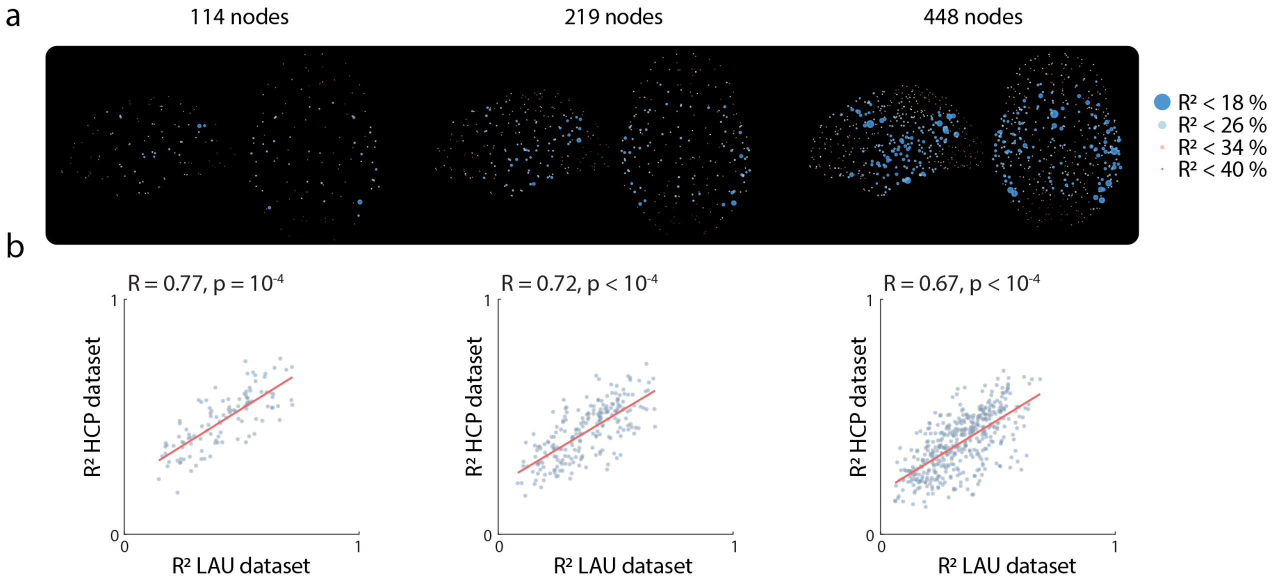

Fig. S2. Replication dataset | To determine whether the results are replicable, we fit the multilinear model in an independently-collected dataset. (a) The spatial distribution of R^2 at 3 different parcellation resolutions. Nodes with smaller structure-function R^2 values are indicated by larger circles and colder colours. (b) The correlation between node-wise R^2 in the discovery dataset (Lausanne dataset; LAU; $N = 40$) and the validation dataset (Human Connectome Project dataset; HCP; $N = 215$).

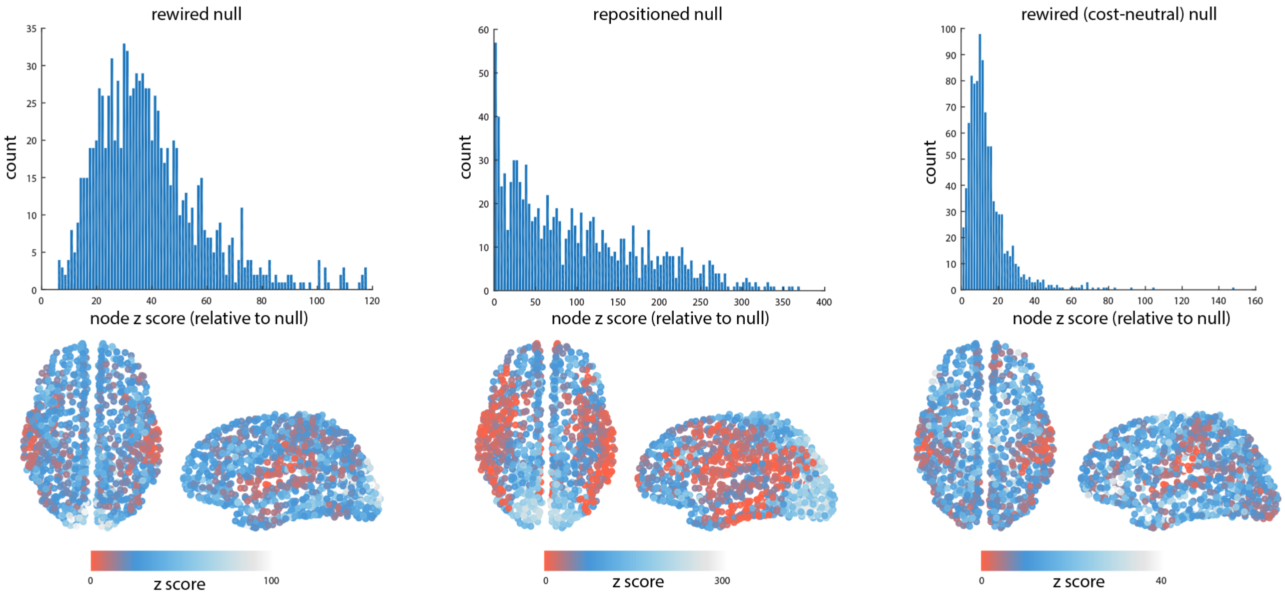

Fig. S3. Benchmarking structure-function R^2 values | Regional structure-function R^2 values were benchmarked against three populations of null or surrogate networks (10,000 repetitions): rewired networks, repositioned networks, and cost-neutral rewired networks. Regional R^2 values were then expressed as z-scores relative to these null distributions. Top: z-scored R^2 values shown in histogram form. Bottom: z-scored R^2 values shown in topographic form. Warm colours indicate lower z-scores, emphasizing nodes with chance-like structure-function correspondence.

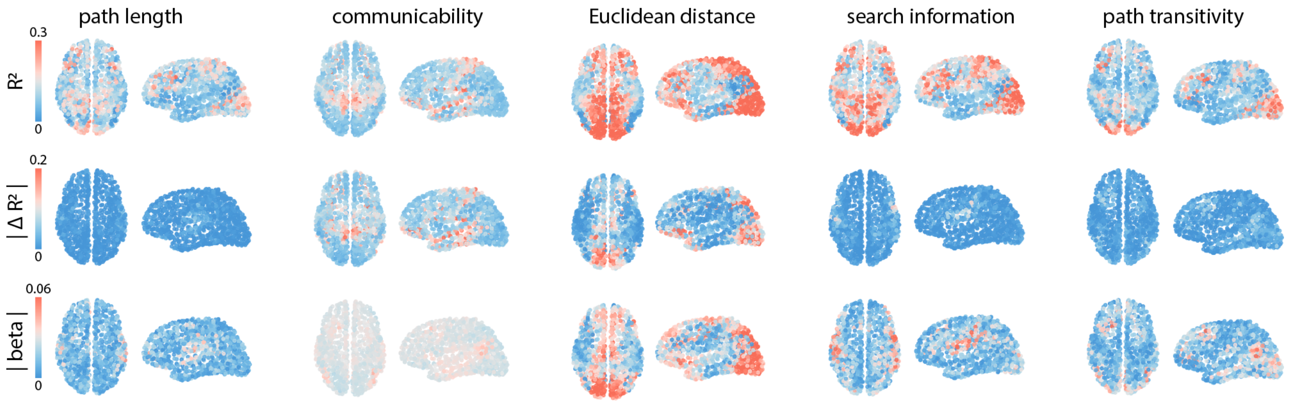

Fig. S4. Variable importance | The contribution of individual independent variables (path length, communicability, Euclidean distance, search information and path transitivity) is assessed using three methods. Top row: Variable importance assessed by single-variable linear regression R^2. Middle row: The structure-function R^2 is first estimated with all variables included in the model, and then re-estimated when individual variables are removed. The contribution of a variable is quantified as the decrease in model fit (ΔR^2) following removal. All values were negative, so absolute values are shown for simplicity. Bottow row: Standardized β values for each predictor when included in the multiple regression model.

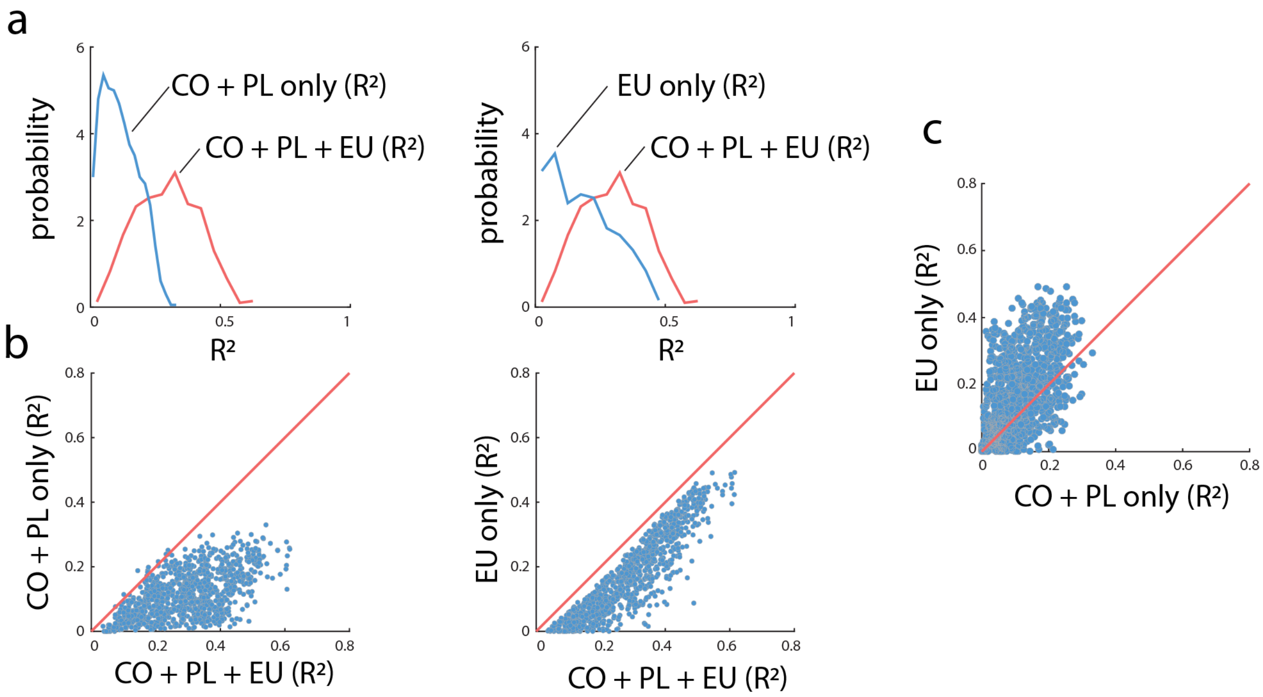

Fig. S5. Disentangling the contributions of topology and geometry | We compare structure-function R^2 for models that incorporate only geometric predictors (Euclidean distance) or only topological predictors (shortest path length and communicability), or both (Euclidean distance, shortest path length and communicability). (a) The distribution of node-specific R^2 values for the original model (red) and for the spatial and topological models (blue). In both cases, removal of the topological/geometrical relationships substantially decreases model fit, shifting the R^2 distribution to the left. (b) R^2 values are plotted for each region (blue), and the identity line is shown as a reference (red). (c) Structure-function R^2 values are plotted for individual regions in the spatial and topological models. EU = Euclidean distance; CO = communicability; PL = path length.

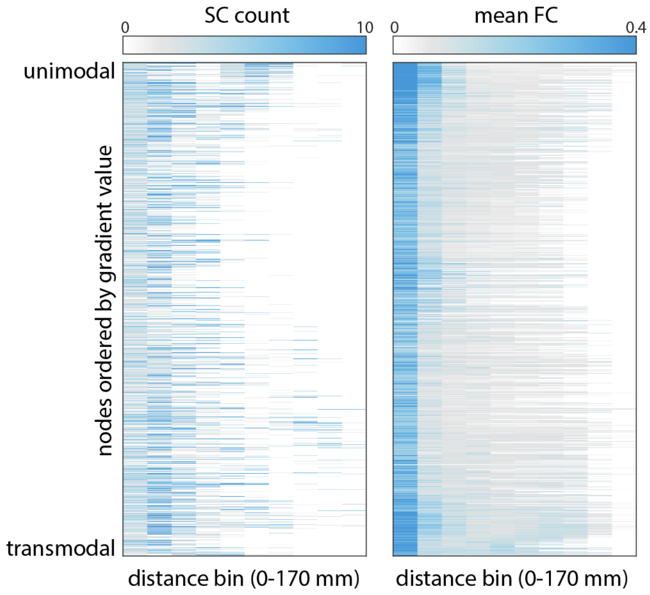

Fig. S6. Regional variation in connection length and density profiles | For each node, we constructed a histogram of connection lengths to other nodes in the network (10 equal width bins, spanning 0 to 170 mm). The histograms are shown for both structural connectivity (the number of structural connections) and functional connectivity (mean correlation). Nodes are ordered by their position along the unimodal-transmodal axis or gradient shown in Fig. 4.
